# Genetic Variants in Nicotine Addiction and Alcohol Metabolism Genes, Oral Cancer Risk and the Propensity to Smoke and Drink Alcohol: A Replication Study in India

**DOI:** 10.1371/journal.pone.0088240

**Published:** 2014-02-05

**Authors:** Devasena Anantharaman, Amélie Chabrier, Valérie Gaborieau, Silvia Franceschi, Rolando Herrero, Thangarajan Rajkumar, Tanuja Samant, Manoj B. Mahimkar, Paul Brennan, James D. McKay

**Affiliations:** 1 Genetic Epidemiology Group, International Agency for Research on Cancer, Lyon, France; 2 Genetic Cancer Susceptibility Group, International Agency for Research on Cancer, Lyon, France; 3 Infections and Cancer Epidemiology Group, International Agency for Research on Cancer, Lyon, France; 4 Prevention and Implementation Group, International Agency for Research on Cancer, Lyon, France; 5 Department of Molecular Oncology, Cancer Institute, Chennai, India; 6 Mahimkar Lab, Advanced Center for Treatment Research and Education in Cancer, Tata Memorial Center, Navi Mumbai, India; Duke Cancer Institute, United States of America

## Abstract

**Background:**

Genetic variants in nicotinic acetylcholine receptor and alcohol metabolism genes have been associated with propensity to smoke tobacco and drink alcohol, respectively, and also implicated in genetic susceptibility to head and neck cancer. In addition to smoking and alcohol, tobacco chewing is an important oral cancer risk factor in India. It is not known if these genetic variants influence propensity or oral cancer susceptibility in the context of this distinct etiology.

**Methods:**

We examined 639 oral and pharyngeal cancer cases and 791 controls from two case-control studies conducted in India. We investigated six variants known to influence nicotine addiction or alcohol metabolism, including rs16969968 (*CHRNA5)*, rs578776 (*CHRNA3)*, rs1229984 (*ADH1B*), rs698 (*ADH1C*), rs1573496 (*ADH7*), and rs4767364 (*ALDH2*).

**Results:**

The *CHRN* variants were associated with the number of chewing events per day, including in those who chewed tobacco but never smoked (P =  0.003, P =  0.01 for rs16969968 and rs578776 respectively). Presence of the variant allele contributed to approximately 13% difference in chewing frequency compared to non-carriers. While no association was observed between rs16969968 and oral cancer risk (OR =  1.01, 95% CI =  0.83– 1.22), rs578776 was modestly associated with a 16% decreased risk of oral cancer (OR =  0.84, 95% CI =  0.72– 0.98). There was little evidence for association between polymorphisms in genes encoding alcohol metabolism and oral cancer in this population.

**Conclusion:**

The association between rs16969968 and number of chewing events implies that the effect on smoking propensity conferred by this gene variant extends to the use of smokeless tobacco.

## Introduction

Cancers of the oral cavity and pharynx contribute to nearly 400,000 new cases each year worldwide, more than half of which occur in India. Each year over 200,000 die of the disease, and over a third of these deaths occur in India [1]. Tobacco use and alcohol consumption are the key established risk factors for oral cancer, with the use of smokeless tobacco being particularly important in the Indian population [2]. Exposure to human papillomavirus is becoming increasingly important to cancers of the oropharynx [3], [4].

Genome-wide association studies (GWAS) have successfully identified disease susceptibility loci to various complex diseases [5]. Lung cancer GWAS and nicotine addiction studies have identified the 15q25 locus harbouring the nicotinic acetylcholine receptor (*CHRN)* gene cluster [6]–[8]. These genes code for receptors expressed in neuronal and other epithelial cells that bind to nicotine and nicotine derivatives [9], [10]. Two *CHRN* receptor subunit variants, rs16969968 and rs578776 have been consistently associated with lung cancer risk and smoking behavior in several populations [11]–[15]. Homozygous carriers of the rs1669968 rare allele have been reported to smoke approximately 1.2 cigarettes more per day [13]. Further, this variant has also been associated with increased risk of Upper Aero-Digestive Tract (UADT) cancer. UADT cancer GWAS and candidate gene association studies have identified genetic variants in the 4q (rs1229984, rs698, rs1573496) and 12q (rs4767364) loci containing genes involved in alcohol metabolism [16], [17]. The balance between alcohol dehydrogenase and aldehyde dehydrogenase activities has been suggested to regulate blood acetaldehyde concentrations that determine the unpleasant symptoms associated with alcohol consumption, thus impacting the ability to consume alcohol and potentially, cancer risk [18], [19]. The alcohol and aldehyde dehydrogenase genes (*ADH and ALDH,* respectively) have been associated with head and neck cancer risk [16], [17], [20]. Although alcohol is an important and established risk factor for oral cancer in India [21]–[24], there is a paucity of data on the association of *ADH* and *ALDH2* variants in this population.

In this study we aimed to (i) determine whether the association between *CHRN* variants and propensity to smoke extends to non-smoking forms of tobacco use (ii) examine if *CHRN* genetic variants influence susceptibility to oral cancer risk in India, (iii) clarify the association between the potential causal variants in the 4q23 (*ADH)* and 12q (*ALDH2)* locus and the risk of oral cancer in India.

## Materials and Methods

### Ethics statement

Written informed consent was obtained from all participants. The IARC multi-center study was approved centrally by the Cancer Institute Ethical Committee at Chennai, India and the overall study was approved by the IARC Ethical Review Committee at the International Agency for Research on Cancer (IARC), Lyon, France. The Mumbai study was approved by the Hospital Ethics Committee of the Tata Memorial Hospital & Cancer Research Institute at Mumbai, India.

### Study description

The present analysis utilized cases and controls from two previously conducted studies in India: the International multicenter oral cancer study (IARC study), and the Mumbai study. The IARC study was coordinated by the International Agency for Research on Cancer (IARC) across nine countries during 1996 to 1999. The study recruited cases and controls from three centers across India, including Bangalore, Chennai and Trivandrum. Cases were histologically or pathologically confirmed primary cancers of the oral cavity and oropharynx. Hospital based controls, one for each case frequency matched for age (5 year period), sex and center were recruited. The overall study participation rate was 93%, details have been described elsewhere [25]–[27]. The Mumbai study was conducted by the Cancer Research Institute, Advanced Center for Treatment, Research and Education in Cancer at the Tata Memorial Center, during 2001 to 2004. Cases were histologically confirmed diagnosis of oral cavity cancers and appropriate controls, frequency matched for age (5 year intervals), gender and tobacco habits were recruited from the Tata Memorial hospital blood bank and nearby dental clinics [28]. Lifestyle and demographic data were collected upon personal interview in both studies.

### Laboratory methods

Genomic DNA was extracted from blood samples using the QIAamp 96 DNA Blood Kit (Qiagen, Hilden, Germany), or with Puregene chemistry (Gentra Systems, Minneapolis, MN, U.S.A.) on an Autopure instrument (Gentra Systems) in the IARC study. In the Mumbai study, DNA was extracted following standard protocol by Sambrook et al [29]. DNA concentrations were measured using PicoGreen double-stranded DNA quantification kits (Molecular Probes, Leiden, The Netherlands). All polymorphisms were analyzed centrally at IARC using the 5’ exonuclease assay. PCR primers and TaqMan probes were synthesized by Applied Biosystems (Foster City, CA). To ensure quality control, DNA samples from case patients and control subjects were randomly distributed on each PCR plate, and all genotyping was conducted by personnel blinded to the case-control status. Ten percent of the study subjects (i.e., both case patients and control subjects), were randomly selected for re-genotyping of each polymorphism to examine the reliability of the genotyping assays, all assays had concordance of >99%. The final analyses included samples that passed stringent quality control (QC) criteria. Subjects were excluded if they failed two or more of the six genotyping assays performed. Further, only cases with >95% genotyping success rate were included. The original IARC study recruited 582 cases and 582 controls from India. Of these, 935 subjects were genotyped, of which 872 passed the described QC criteria and were included in the present analysis. In the Mumbai study, of the 576 subjects genotyped, 226 cases and 332 controls cleared the described QC and were included in this analysis. A total of 639 cases and 791 controls were included in this analysis following exclusions described above.

### Statistical methods

Data on alcohol consumption, tobacco use, chewing and smoking were collected in both the participating studies. The definition of drinker, smoker and chewer were consistent across both studies and included individuals who responded confirming ever use of alcohol, ever smoking any tobacco product (cigarette, cigar, pipe, hooka, cigarette and/or bidi), ever use of any smokeless tobacco product with or without betel quid. Several forms of smokeless tobacco are consumed in India, while *khaini, mawa, mishri, zarda, gudakhu* and *gutkha* are all formulations containing tobacco with different flavoring agent*s, pan masala* is an areca nut product that does not contain tobacco, to which tobacco may be added externally. Duration and frequency of exposure to tobacco and alcohol were calculated in each study as described previously [25]–[28]. Briefly, smoking duration was calculated as the difference between age at start and age at stop of smoking, taking into account overlapping periods of various types of smoking (except in the Mumbai study where only cigarette and bidi use was recorded, and very few overlapping exposures were observed). Smoking frequency was calculated as number of cigarette equivalents smoked per day, with 1 cigar =  4 cigarettes, 1 pipe =  5 cigarettes and 1 bidi =  0.5 cigarettes. Chewing included use of smokeless tobacco products, including betel quid, areca nut with &/or without tobacco and *pan masala* in the IARC study, and tobacco with lime, *pan masala, khaini, mawa, mishri, zarda, gudakhu, snuff* and *gutkha* in the Mumbai study. Chewing frequency was calculated as the cumulative number of chewing events per day, taking into account overlapping periods of exposure. For the calculation of lifetime alcohol consumption, the percentage of ethanol was estimated as 40% for spirits (whisky, gin, rum, brandy, arrack and country liquor), 3% for beer and 5% for toddy (except in the Mumbai study). Smoking, chewing and alcohol related oral cancer risk was calculated using logistic regression models adjusted for age, sex, level of education and center. The corresponding attributable fractions were calculated based on the formula: p (ec)x(OR-1)/OR; where p(ec) is the proportion of exposed among cases [30], [31]. Chi-square tests were performed to compare allele frequencies observed in the present study with our previously published results [16], [17]. The associations between the genetic variants and oral cancer were estimated by logistic regression adjusted for age, sex and center and tobacco use where appropriate. The association between the *CHRN* variants and propensity to use tobacco was assessed using linear regression models with log transformed number of cigarette equivalents smoked per day, or number of chewing events per day as the outcome variable. Results represent the fold-change in the number of cigarettes smoked per day or the number of chewing events per day for each copy of the rare allele. These analyses were adjusted for age, sex, center and case-control status. Similar analyses were performed to examine the association between ADH/ALDH2 variants and the propensity to drink alcohol, measured as number of drinks of ethanol consumed per day. All statistical analyses were performed using STATA version 11 (STATA Corp, College Station, Tx), and the reported p-values are two sided.

## Results

Table 1 describes the characteristics of the study population. Of the 639 cases and 791 controls included in the present analysis, the IARC international study contributed the largest proportion of subjects (61%). Majority of the cases were male (65%) and had completed lower levels of education and were cancers of the oral cavity (96%). Tobacco smoking and chewing were associated with increased risk of oral cancer (OR =  5.23, 95% CI =  3.39– 8.07 and OR =  8.30, 95% CI =  5.78– 11.93, respectively), and the combined use of smoking and smokeless tobacco products conferred a 12-fold increased risk of oral cancer (OR =  12.40, 95% CI =  7.80– 19.70). Ever use of alcohol was associated with a 1.67-fold increased risk of oral cancer (OR =  1.67, 95% CI =  1.25– 2.22).

**Table 1:** Description of study group.

Description	Controls	Oral cancer			
	(n = 791)	(n = 639)			
	N (%)	N (%)			
**Study**					
ACTREC study	332 (42.0)	226 (35.4)			
International oral cancer study	459 (58.0)	413 (64.6)			
**Gender**					
Male	510 (64.5)	383 (59.9)			
Female	281 (35.5)	256 (40.1)			
**Age groups**					
< = 45 years	333 (42.1)	162 (25.4)			
46– 55 years	199 (25.2)	190 (29.7)			
56–65 years	178 (22.5)	195 (30.5)			
>65 years	81 (10.2)	92 (14.4)			
**Years of education**					
<5 years	203 (26.8)	271 (44.4)			
5–7 years	326 (43.0)	227 (37.2)			
>7– 12 years	141 (18.6)	86 (14.1)			
>12–15 years	67 (8.8)	23 (3.8)			
>15 years	21 (2.8)	4 (0.7)			
**Tobacco use**			**OR (95%CI)** ?	**p-value**	**AF** $
Never tobacco	308 (39.0)	73 (11.5)	1.0		
Smoking only	150 (19.0)	118 (18.6)	5.23 (3.39– 8.07)	<0.0001	15
Chewing tobacco only	250 (31.7)	326 (51.4)	8.30 (5.78– 11.93)	<0.0001	45
Both	81 (10.3)	117 (18.5)	12.40 (7.80– 19.70)	<0.0001	17
**Alcohol consumption**					
Never	608 (76.9)	468 (73.9)	1.0		
Ever	183 (23.1)	165 (26.1)	1.67 (1.25– 2.22)	0.001	7
**Cancer Site**					
Oral cavity		613 (96.0)			
Pharynx		26 (4.0)			

? adjusted for age, sex, level of education and center.

$ Attributable fraction, expressed as percentage. Calculated using the formula p(ec)x (OR-1)/OR, where p(ec) is the proportion of exposed among cases.

The distribution of all six variants examined in the present analysis was consistent with expectations of genotype distributions based on the Hardy-Weinberg principle. The allele frequencies were similar across the participating studies (data not shown). We found that the variants in alcohol dehydrogenase genes- rs1229984 (*ADH1B)*, rs698 (*ADH1C)* and rs1573496 (*ADH7)* and the nicotine dependence gene variant rs16969968 were less common among Indians compared to previous studies in predominantly western populations. On the other hand, rs578776 was more common among Indians (table2).

**Table 2:** Variants examined in the present study.

Gene	SNP	Minor allele	Frequency (%)		p-heterogeneity*
			Present study	Published studies [ref]	
*ADH1B*	rs1229984	T	0.02	0.06 [17]	<0.01
*ADH1C*	rs698	C	0.33	0.44 [17]	<0.01
*ADH7*	rs1573496	C	0.04	0.11 [17]	<0.01
*ALDH2*	rs4767364	A	0.66	0.30 [17]	<0.01
*CHRNA3*	rs578776	A	0.51	0.40 [8]	<0.01
*CHRNA5*	rs16969968	A	0.18	0.34 [8]	<0.01

* indicates heterogeneity of minor allele frequencies between the present study and previous European studies, minor allele calling was similar between Indians and Europeans.

We examined the association between *CHRN* variants and the propensity to use tobacco; the results are summarized in table 3. The carriers of the rs16969968 rare allele (A) showed some tendency to smoke more, although were not statistically significant. We also observed an increase in the propensity to chew tobacco with the rs16969968 variant (p =  0.03). Presence of each copy of the A allele corresponded to an increase by 13% more chewing events per day (fold-change =  1.13, 95% CI =  1.01– 1.25) compared to non-carriers. The association with chewing remained robust upon further adjustment for smoking frequency (fold-change =  1.13, 95% CI =  1.01– 1.26) and the association was consistent among never smokers who chewed tobacco (p =  0.003) (fold-change =  1.19, 95% CI =  1.06– 1.34). We observed a similar decrease in the propensity to chew among carriers of the rs578776 rare variant (fold-change =  0.91, 95% CI =  0.84– 0.99), the association being stronger among chewers who never smoked.

**Table 3:** *CHRN* variants and propensity to smoke or chew tobacco.

Adjustment model	n	Fold-change in tobacco use/day (95% CI)	P- Value
**Smoking propensity** ? (Cigarettes/day)			
**rs16969968 (CHRNA5)**			
Base model$	388	1.13 (0.96– 1.34)	0.15
Base+ chewing frequency	388	1.13 (0.95– 1.34)	0.16
Base, among never chewers only	212	1.19 (0.98– 1.44)	0.08
**rs578776 (CHRNA3)**			
Base model$	383	0.99 (0.86– 1.13)	0.87
Base+ chewing frequency	383	0.99 (0.86– 1.13)	0.87
Base, among never chewers only	205	0.95 (0.82– 1.11)	0.54
Propensity to chew tobacco* (Chewing events/day)			
**rs16969968 (CHRNA5)**			
Base model$	622	1.13 (1.01– 1.25)	0.03
Base+ smoking frequency	622	1.13 (1.01– 1.26)	0.03
Base, among never smokers only	446	1.19 (1.06– 1.34)	0.003
**rs578776 (CHRNA3)**			
Base model$	613	0.91 (0.84– 0.99)	0.04
Base+ smoking frequency	613	0.91 (0.84– 0.99)	0.04
Base, among never smokers only	435	0.88 (0.81– 0.97)	0.01

? derived from linear regression of log transformed number of cigarettes smoked per day as outcome, respective *CHRN* variant as explanatory.

* derived from linear regression of log transformed number of chewing events per day as outcome, respective *CHRN* variant as explanatory.

$ Base model included age, sex, center and case-control status.

significant p- values are indicated in bold.

**Figure 1:**
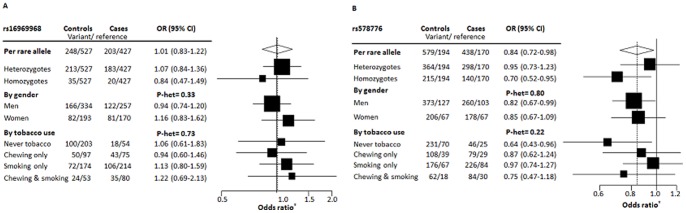
*CHRN* variants and risk of oral cancer. Panel A shows the association between rs16969968 and oral cancer; Panel B shows the association between rs578776 and oral cancer. Forest plot represents odds ratios derived from the log additive multivariate model adjusted for age, sex, level of education and center, as appropriate.

No association was observed for rs16969968 and oral cancer risk in this study overall (OR =  1.01, 95% CI =  0.83– 1.22), by gender (p-heterogeneity =  0.33). Although no effect modification by smoking or chewing status was observed, a statiscally non-significant oddds ratio of 1.22 (95% CI =  0.69– 2.13) was observed in chewers and smokers (figure 1). We observe little evidence for a statistical interaction between the rs16969968 variant and tobacco use in the risk of oral cancer (OR: 1.18, 95% CI: 0.68– 2.07). On the other hand, rs578776 was associated with decreased risk of oral cancer in this study (OR =  0.84, 95% CI =  0.72– 0.98). A gene-dose effect was observed for carriers of the rare allele (p-trend =  0.02). Although no heterogeneity was observed based on tobacco habits, a decreased risk was also observed among never smokers and never chewers (OR =  0.64, 95% CI =  0.43–0.96) (figure 1).

We examined variants in genes related to alcohol metabolism since they were strongly associated with head and neck cancer in previous studies. None of the four variants (rs1229984, rs698, rs1573496, and rs4767364) examined, showed any association with oral cancer risk (table 4). We also performed restricted analyses among ever drinkers given the important effect modification by alcohol consumption. Although the lack of association remained consistent, the observed estimates followed the direction anticipated based on expectations from previous GWAS and candidate gene studies. Stratified analyses based on gender did not reflect any heterogeneity in the observed lack of associations (table 4). Further, none of the genetic variants in alcohol metabolism genes appeared to influence alcohol consumption levels (Table S1).

**Table 4:** Association between *ADH* & *ALDH2* variants and risk of oral cancer.

Variant	Controls	Oral cancer cases	
	(variant/reference)	OR (95%CI)*	
**rs1229984 (** ***ADH1B)***			
Per rare allele	29/744	22/596	0.97 (0.52– 1.79)
By alcohol status (p-het = 0.98)			
Never drinker	23/570	19/432	1.08 (0.55– 2.13)
Ever drinker	6/174	3/159	1.11 (0.22– 5.54)
**By sex (p-het = 0.37)**			
Men	20/480	11/362	0.80 (0.36– 1.76)
women	9/264	11/234	1.49 (0.49– 4.51)
**rs698 (** ***ADH1C)***			
Per rare allele	419/343	314/276	0.97 (0.82– 1.16)
**By alcohol status (p-het = 0.95)**			
Never drinker	325/264	230/181	0.98 (0.80– 1.20)
Ever drinker	94/79	81/66	0.96 (0.66– 1.41)
**By sex (p-het = 0.46)**			
Men	265/217	188/149	0.99 (0.80– 1.23)
women	154/126	126/100	0.86 (0.62– 1.19)
**rs1573496 (** ***ADH7)***			
Per rare allele	56/721	48/581	1.18 (0.77– 1.81)
**By alcohol status (p-het = 0.65)**			
Never drinker	45/44	37/36	1.09 (0.67– 1.76)
Ever drinker	11/169	10/152	1.42 (0.51– 3.98)
**By sex (p-het = 0.84)**			
Men	37/36	28/28	1.16 (0.68– 1.96)
women	19/256	20/232	1.27 (0.58– 2.78)
**rs4767364 (** ***ALDH2)***			
Per rare allele	671/101	537/82	0.92 (0.78– 1.09)
**By alcohol status (p-het = 0.04)**			
Never drinker	521/249	386/206	0.84 (0.69– 1.02)
Ever drinker	150/72	146/71	1.27 (0.89– 1.81)
**By sex (p-het = 0.07)**			
Men	421/204	326/169	1.03 (0.84– 1.26)
women	250/117	211/108	0.72 (0.52– 1.00)

* Odds ratios were derived from log additive model adjusted for age, sex, tobacco exposures and center.

P indicates p-value for heterogeneity.

## Discussion

The *CHRN* variants, rs16969968 and rs578776, have been associated with nicotine addiction and lung cancer risk and these associations appear to be independent [15], [32]. Here, we demonstrate that the association between *CHRN* variants also extends to the propensity to chew tobacco, a relevant oral cancer risk factor in the Indian population.

Several formulations of smokeless tobacco are commercially available and commonly used in India; these were grouped together in this study. The number of chewing events per day was recorded in both studies included in this analysis; however it is important to note that this does not reflect the amount of product chewed each time and hence, constitutes a potential source of misclassification of exposure. Given that we observe a measurable association with such a crude estimate implies that the true effect of the *CHRN* variants and chewing dependence could be potentially higher than that observed in the present study. Previous studies have demonstrated that UADT cancer risk associated with rs16969968 is higher among women and for cancers of the oral cavity and larynx [13], [33]. In this study, the increased propensity to chew tobacco did not translate to an increased risk of oral cancer for rs16969968; although rs578776 was associated with both decreased chewing propensity and a modest decrease in oral cancer risk. Interestingly, rs578776 was also associated with decreased oral cancer risk in never tobacco users, albeit based on small numbers. This association may be influenced by misclassification of exposure or potentially through a tobacco independent effect. Larger focused studies will be required to address this issue.

The *ADH* genes, correlated as a consequence of linkage disequilibrium, are located in the 4q23 locus. Three alleles, rs1229984, rs698 and rs1573496 within the *ADH* locus have been associated with UADT cancer risk in Europeans and other Asian populations [17], [34]–[36]. While there has been some evidence to suggest *ADH* variants may influence oral and neck cancer risk in India, in particular rs698 [37], we observed no association between rs1229984, rs698 and oral cancer risk. Similarly the *ALDH2* variant rs4767364, identified through a recent UADT GWAS [17], was not associated with oral cancer in this study. The fraction of alcohol consumers in this study was comparable to other reports from this population, but is notably lower than reported among Europeans [38], [39]. Further, the rarity of the risk alleles in the Indian population suggests that the contribution of alcohol and alcohol related variants to oral cancer susceptibility in India is likely to be limited.

To the best of our knowledge, we report for the first time that the association between 15q25 locus and propensity to smoke extends to tobacco chewing. We also provide suggestive evidence that 15q25 region containing the *CHRN* gene cluster may contribute to oral cancer susceptibility in India. In this study, rs16969968 variant was associated with increased propensity to chew tobacco, and rs578776 was associated with decreased chewing propensity and oral cancer risk. Larger studies will be required to clarify the extent of risk modification mediated by propensity to use tobacco. This study also indicates that variants in alcohol metabolizing genes appear to be less important to oral cancer susceptibility in India. These data reiterate the importance of disease etiology, the relative contribution of risk factors and their impact in replication of GWAS identified risk loci across populations.

## Supporting Information

Table S1 **Variants in alcohol metabolizing genes and alcohol consumption levels.** Fold-change were derived from linear regression models adjusted for age, sex, center and case-control status. Log transformed number of alcoholic drinks per day was treated as the outcome variable for each of the respective genetic variant as the explanatory.
(DOCX)
