# Supplementary material for: Genetic Variants in Nicotine Addiction and Alcohol Metabolism Genes, Oral Cancer Risk and the Propensity to Smoke and Drink Alcohol: A Replication Study in India
Source: PLoS One. 2014 Feb 5;9(2):e88240. doi: 10.1371/journal.pone.0088240 (PMC3914962; doi:10.1371/journal.pone.0088240)
Supplement: Table S1 — Variants in alcohol metabolizing genes and alcohol consumption levels. Fold-change were derived from linear regression models adjusted for age, sex, center and case-control status. Log transformed number of alcoholic drinks per day was treated as the outcome variable for each of the respective genetic variant as the explanatory. (DOCX) [file pone.0088240.s001.docx]

**Table S1: Variants in alcohol metabolizing genes and alcohol consumption levels**

| **Genotype** | **N** | **Fold-change in drinks/ day (95% CI)** | **p-value** |  |
| --- | --- | --- | --- | --- |
| rs1229984 (*ADH1B)* | 340 | 1.00 (0.72- 1.40) | 0.99 |  |
| rs698 (*ADH1C)* | 335 | 1.03 (0.95- 1.12) | 0.46 |  |
| rs1573496 (*ADH7)* | 340 | 1.00 (0.81- 1.24) | 0.99 |  |
| rs4767364 (*ALDH2)* | 337 | 1.04 (0.96- 1.12) | 0.31 |  |
| ^^^ derived from linear regression adjusted for age, sex, center and case-control status. Log transformed number of alcoholic drinks per day was treated as the outcome variable for each of the respective genetic variant as the explanatory | | | | |
